# Temporal dynamics of EEG activity during short- and long-wavelength light exposures in the early morning

**DOI:** 10.1186/1756-0500-7-113

**Published:** 2014-02-26

**Authors:** Yosuke Okamoto, Mark S Rea, Mariana G Figueiro

**Affiliations:** 1 Lighting Research Center, Rensselaer Polytechnic Institute, 21 Union Street, Troy, NY 12180, USA; 2 Present Address: Health Research Institute, National Institute of Advanced Industrial Science and Technology (AIST), 1-8-31 Midorigaoka, Ikeda, Osaka 563-8577, Japan

**Keywords:** Light exposure, Monochromatic light, Alertness, EEG, Alpha activity

## Abstract

**Background:**

It is well known that exposure to light, especially of short wavelength, enhances human alertness during the nighttime. However, more information is needed to elucidate the effects of light wavelength on alertness at other times of day. The present study investigated how two narrowband light spectra affected human alertness during the morning after awakening. We measured electroencephalography (EEG) during 48-minute exposure to narrowband short- and long-wavelength light and darkness in the early morning.

**Results:**

Power densities of EEG during each light exposure were calculated. The time course of EEG power indicated that, compared with remaining in darkness, the power in the alpha frequency range (8–13 Hz) was significantly lower after approximately 30 minutes of exposures to both the short- and the long-wavelength light.

**Conclusions:**

These results suggest that not only short-wavelength light but also long-wavelength light, which does not suppress melatonin levels at night, can affect alertness in the early morning. These results suggest that the alerting effects of light in the early morning hours may be mediated by mechanisms other than those that are exclusively sensitive to short-wavelength light.

## Background

Previous studies have shown that ocular light exposure during the night enhances human alertness. Nocturnal exposure to bright white light (>2500 lux at the cornea) reduces subjective sleepiness, increases task performance, increases body temperature and heart rate, reduces low frequency (theta and alpha ranges) and increases high frequency (beta range) electroencephalographic (EEG) activity, reduces the incidence of slow-eye movements (SEMs), and suppresses melatonin, relative to dim light exposure [1-7]. Some studies have suggested that measurements of the effects of light on alertness are highly correlated with melatonin suppression [6,8]. It is now well known that nighttime melatonin secretion in humans is effectively suppressed by short-wavelength (blue) light [9-11] and is not affected by low levels of long-wavelength (red) light. However, Figueiro *et al.* have shown that exposure to both short- (λ_max_ = 470 nm at 40 lux) and long-wavelength (λ_max_ = 630 nm at 40 lux) light during the nighttime reduced alpha power and increased beta power in EEG activity relative to a preceding dark condition, even though only the short-wavelength light suppressed melatonin [12]. Figueiro and Sahin also showed that the same long-wavelength light that increased alertness at night was effective at increasing alertness in the middle of the afternoon when melatonin levels are low, as shown by a reduction in alpha, alpha-theta and theta power in EEG activity [13]. Thus, their results suggest that nocturnal melatonin suppression is not needed for the light-induced alertness during the nighttime.

Most studies of the effects of light exposure on alertness have been performed during the nighttime, because the effects of light can be more easily observed at this time, when human alertness levels are low. In fact, earlier studies investigating the impact of daytime light exposure on alertness, when melatonin levels are low, have shown no difference in the EEG activity following bright white light exposure relative to dim light exposure [2,14]. However, more recently, one study of the impact of daytime exposure to a high level of white light (1000 lux) compared with a low light level control condition (<5 lux) reported that subjective sleepiness, the incidence of SEMs, and reaction times measured in sleep-restricted subjects were all reduced by bright light exposure, even though melatonin levels were no different for the two light conditions [15].

Since our understanding of the impact of light on brain functions is somewhat limited, more information is clearly needed to elucidate the effects of light on alertness during the day and whether light can affect human alertness independent of melatonin suppression. The present study sought to examine how light affect human alertness during the morning after awakening, when melatonin levels would be naturally decreasing. Two narrowband light sources were used; the short-wavelength (blue) light (λ_max_ = 470 nm at 40 lux) chosen for the study had been previously shown to reliably suppress nocturnal melatonin, whereas the long-wavelength (red) light (λ_max_ = 630 nm at 40 lux) did not [16]. EEG activity was measured during 48-minute exposure to each of these lights and during a dark condition in the early morning after awakening. It was reasoned that if the short- and long-wavelength lights both affected EEG activity in the morning relative to the dark condition, then additional evidence would have been acquired indicating, first, that light can affect alertness during the early morning, and, if true, that melatonin, because it is decreasing naturally, is not needed to modulate levels of alertness. It has been shown that an increase in power in the low frequency range including theta and alpha frequency ranges of EEG activity obtained while subjects are awake and with the eye open is associated with sleepiness [17-20]; moreover, exposure to light during the nighttime has been associated with reductions of EEG activity in the low frequency range [4-6,12,16,21]. Therefore, we hypothesized that EEG power levels in the theta and alpha ranges would be reduced after a 48- minute exposure to the short- and long-wavelength lights but not after exposure to darkness.

## Methods

### Subjects

Nine adults (five males and four females) participated in the study. Age ranged from 22 to 34 years (mean age 26.4 ± 4.2 years). All subjects had normal or corrected-to-normal vision. Absence of color blindness was assessed by the 38-plate edition of Ishihara’s Test for Color Blindness. None of the subjects were smokers, and all were instructed to refrain from consuming caffeine and alcohol during the 12-hour period preceding the experiments. Subjects were asked to keep a regular schedule and go to sleep at their usual bedtime the night before the experiments. This study was approved by the Institutional Review Board at Rensselaer Polytechnic Institute (Troy, NY, USA). All subjects signed the approved consent form and were paid US$40 per session to participate in the study.

### Light exposure

Two narrowband lights were presented to subjects with natural pupils via light-integrating boxes. Light emitting diode (LED) arrays (iCove; Color Kinetics, Boston, MA, USA) were mounted inside the front face of the box and the inside of the boxes was painted matt white. A uniform and non-glaring distribution of light was created within the box. A chinrest was mounted near the front of the box. The spectral properties of the short- and long-wavelength lights were confirmed with a spectroradiometer (PR-705; Photo Research, Chatsworth, CA, USA). The peak wavelength and the full width at half maximum (FWHM) for the short- and long-wavelength lights were 470 nm with an FWHM of 25 nm and 630 nm with an FWHM of 25 nm, respectively. The illuminance levels of both lights were measured with an illuminance meter (X9-1; Gigahertz-Optik, Puchheim, Germany), and set at 40 lux at the subjects’ corneas (40 μW/cm^2^ and 19 μW/cm^2^ for the short- and long-wavelength lights, respectively). The single green-LED as a fixation point was lit at a very low light level so that the illuminance level of the no-light condition was < 0.01 lux at the subjects’ cornea.

### EEG recording and analysis

EEG was recorded from four Ag-AgCl electrodes placed at Fz, Cz, Pz and Oz according to the International 10–20 system. We used a BioSemi ActiveTwo system (Biosemi, Amsterdam, The Netherlands) for EEG recording. In this system, common mode sense active electrode and driven right leg passive electrode were used to form a feedback loop driving the average potential of the subject as closely as possible to the amplifier zero. These electrodes were placed on the forehead. The reference electrodes were placed on the left and right ear lobes. The electro-oculogram (EOG) was recorded from an electrode placed directly below the right eye to monitor blinking. EEG and EOG data were digitized with a sampling rate of 2048 Hz. The EEG and EOG data were re-referenced off-line to the averaged ear lobes and band-pass filtered between 0.3 and 40 Hz.

### Protocol

This study was conducted over several weeks in January and February. The experiment consisted of three different sessions, separated by at least 1 week. All sessions started at approximately 07:00 hours and ended approximately at 08:00 hours. Subjects were asked to come to the laboratory 30 minutes prior to the start of the experimental sessions. All data were collected during the winter months; therefore, subjects came to the laboratory before daybreak and were not exposed to daylight on the way to the laboratory. Any other light exposure prior to coming to the laboratory would have been similar in all three experimental sessions because subjects were asked to come to the laboratory soon after waking. We asked the subjects about their usual wake-up time and the time when they awoke on the days of the experiments. On average, subjects woke up approximately 90 minutes earlier than the usual wake-up time on days between the experimental sessions. Although subjects woke earlier than usual for the experiment, there was no significant difference in the deviation of wake-up time from the usual across the three light conditions (*p* = 0.88).

**Figure 1:**
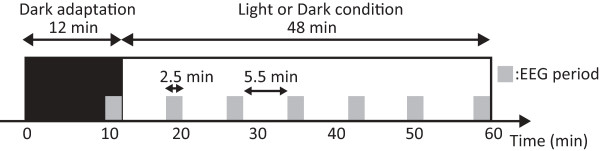
**Experimental design.** Gray rectangles indicate EEG measurement periods. EEG measurements of 150 seconds were conducted seven times within a session.

Each session was conducted on a different day for different subjects; subjects completed three sessions on three successive weeks. Each subject came back to the laboratory during three consecutive weeks to complete the experimental protocol. In each session (Figure 1), a subject first sat in front of an unenergized (dark) light box for 12 minutes. Subjects were asked to look inside the boxes at all times and keep their eyes open for the duration of the experiment. Subjects were instructed to place their chins on a chinrest prior to presentation of a short tone for 5 seconds, followed by initiation of the EEG recordings. To minimize eye movements during the EEG interval, subjects were instructed to fixate on the single green LED placed on the back wall of the light box. Subjects were advised to refrain from head and body movements as much as possible to prevent motion-related interference with the EEG signal. In between each EEG interval, subjects were instructed to remove their chins from the chinrest and relax while keeping their head aligned with the boxes to assure proper light exposures.

EEG measurements were obtained for 2.5 minutes at the end of the dark period. After the first 12 minutes, one of the three light conditions (short-wavelength light, long-wavelength light, or dark) was presented for 48 minutes; the order of the light conditions was counterbalanced across the subjects. Six EEG measurements of 2.5 minutes were obtained separated by 5.5-minute intervals. Short auditory tones signaled the beginning and end of the EEG recording periods.

### Data analyses

The EEG data during the EEG period (150 seconds) were extracted and divided into 1-second epochs with 0.5-second epoch overlap. Epochs contaminated by peak-to-peak potentials over 30 μV or accompanied by an EOG exceeding 50 μV were rejected from further analysis. In total, 61% of all epochs remained in the processing data. These epochs were passed through a 10% cosine window and subjected to fast Fourier transform (FFT) analysis. FFT power spectra for the epochs were averaged in an EEG data collection period. The spectral power values were summed within the following frequency ranges: theta (4–7 Hz) and alpha (8–13 Hz). EEG data derived from the electrodes Fz and Cz were used for the calculation of the theta power, and those from Pz and Oz were used for the alpha power.

To investigate temporal variations in EEG activities, time courses of EEG power in the theta and alpha frequency ranges were independently analyzed. The ratio of EEG power at each interval relative to the power during the dark adaptation period (i.e., the first EEG interval) was calculated for each experimental session. In each frequency range (theta and alpha), a 3 × 7 (3 light conditions × 7 time interval) repeated measures analysis of variance (ANOVA) were performed. Two-tailed *post hoc* Student’s *t*-tests with Bonferroni correction were performed to investigate the significant main effects and interactions. When the interaction reached a significant level, *t*-tests were conducted to assess the effect of light condition at each time interval. The significance level was set at *p* < 0.05.

## Results

**Figure 2:**
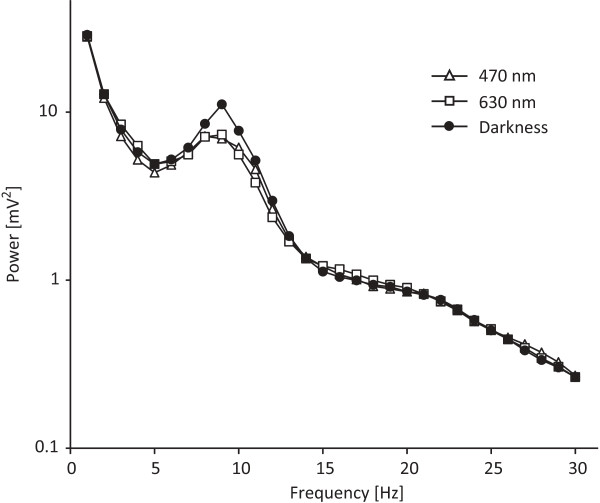
**The EEG power spectra for exposure to short- (circles) and long-(squares) wavelength lights, and darkness (triangles).** Data averaged for all subjects, and EEG measurement periods collected during the last 48 minutes (i.e., EEG intervals 2–7) were included.

**Figure 3:**
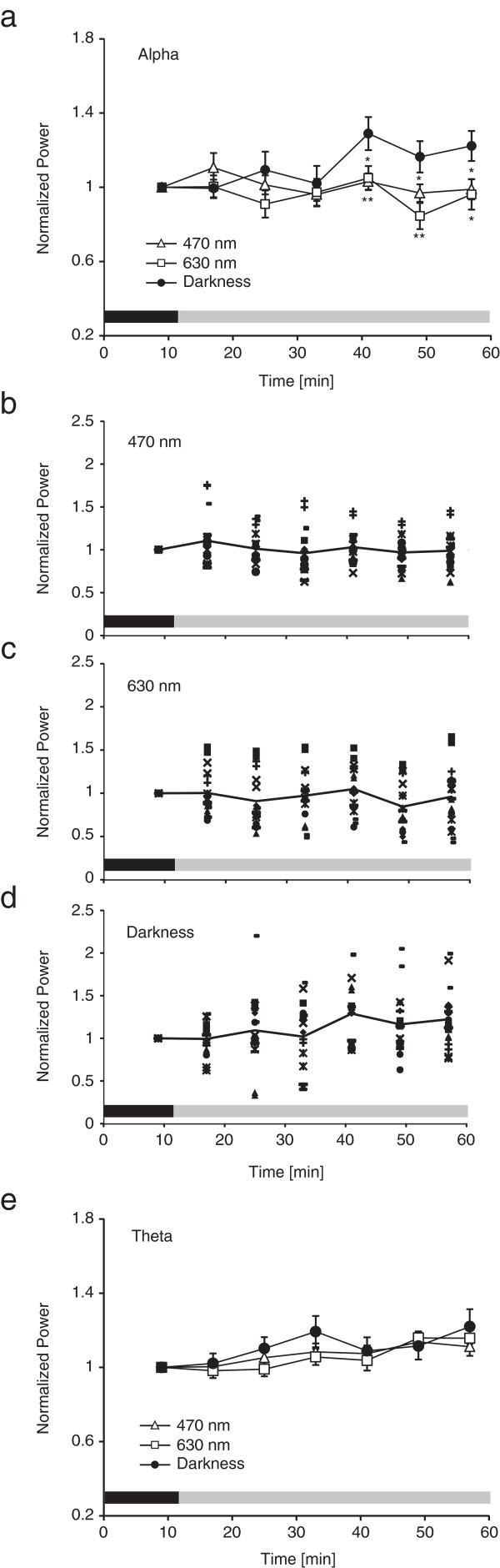
**Time courses of EEG power in the three light conditions. (a)** Grand average time courses of EEG power in the alpha range for exposure to short- (circles) and long- (squares) wavelength lights, and darkness (triangles). Black and gray bars indicate the dark adaptation period and the light or dark exposure period, respectively. Error bars indicate ±1 SEM. Asterisks show significant differences compared with the darkness (*p < 0.05, **p < 0.01). Individual EEG power values in the alpha range in the **(b)** short-wavelength light, **(c)** long-wavelength light and **(d)** dark conditions. The same symbol indicates the values obtained from the same subject. **(e)** Grand average time courses of EEG power in the theta range.

Figure 2 shows the EEG power spectrum averaged over all EEG intervals in the 48-min light period (i.e., EEG intervals 2–7). EEG power in the theta frequency range was derived from Fz and Cz channels and in the alpha range was derived from the Pz and Oz channels. The 3 × 7 repeated-measures ANOVAs are summarized in Table 1. Results showed that there was no significant main effect of light condition for power in the alpha and theta ranges. A significant interaction was found for the alpha power (*p* < 0.05). Student’s *t*-tests revealed that the EEG alpha power in the dark condition was significantly greater than that in the short- and long-wavelength light conditions at approximately 30, 40 and 50 minutes after light exposure (i.e., in EEG intervals 5–7), as shown in Figure 3. The correlation between the normalized EEG alpha power averaged throughout the experiment except for the dark adaptation period (i.e., EEG intervals 2–7) and the deviance of the subjects’ wake up time on the day of the experiment from their usual wake-up time was analyzed for each lighting condition. The Pearson’s correlation coefficients were -0.31 (*p* = 0.22), -0.38 (*p* = 0.12) and 0.59 (*p* < 0.01) for the short-wavelength light, long-wavelength and dark conditions, respectively, suggesting that alpha power increased as the deviance from the usual wake-up times increased only in the dark condition.

**Table 1:** Results of 3 × 7 repeated measures ANOVAs for EEG power in the three frequency ranges

**Frequency range**	**Factor**	**df**	**F-ratio**	**p-value**
Theta	Light condition	2	0.43	0.65
	Time interval	6	9.82	< 0.01
	Light condition and time interval	12	1.19	0.29
Alpha	Light condition	2	1.41	0.25
	Time interval	6	3.58	< 0.01
	Light condition and time interval	12	2.71	< 0.01

df: degree of freedom.

For power in the theta range, the ANOVAs revealed a significant main effect of time interval (*p* < 0.01). Student’s *t*-tests showed that power was significantly greater at approximately 30, 50 and 60 minutes after the experiment began (i.e., in EEG intervals 4, 6 and 7) relative to the first 20 minutes into the experiment (i.e., in EEG intervals 1 and 2) (*p* < 0.05).

## Discussion

The present results showed that EEG power in the alpha frequency range was significantly reduced after approximately 30 minutes of morning exposure to both the short- and long-wavelength lights compared with darkness. It has been previously shown that EEG power in the alpha and theta frequency ranges increases with sleepiness under eyes-open conditions [17-20]. Moreover, the alerting effects of bright white light during the night were demonstrated by a reduction of EEG activity in the theta-alpha frequencies [4,6], as shown by other correlates of alertness. Although the effects of light wavelength were not observed in the theta range, taken together with these previous findings, the present results suggest that 30-minute exposure to both short- and long-wavelength light can increase one measure of alertness in the early morning. Moreover, EEG alpha power throughout the dark condition correlated with the deviance in wake-up time on the day of the experiment from the usual wake-up time, whereas alpha power under the light conditions did not. This implies that over the time course of session, the earlier the subjects woke up with respect to their usual wake times, the sleepier they felt during the dark session but not during the two light.

The light-induced alerting effects observed in nighttime experiments may have been a result of the hormone melatonin, which is believed to promote sleepiness in diurnal species. However, daytime light exposure, when melatonin is low, can also reduce subjective sleepiness and improve task performance, compared with a dim light control condition [15]. In addition, a decrease in EEG power in theta and alpha ranges has been observed after exposure to long-wavelength light in the daytime [13]. Although we did not measure melatonin levels in the present study, short- and long-wavelength light exposure in the morning decreased power in the alpha frequency range while subjects were awake and with eyes opened, at a time when melatonin levels would have been naturally decreasing. These findings suggest that melatonin suppression alone is not the only mechanism mediating the alerting effects of light. As support for this suggestion, Figueiro and colleagues showed that exposure to long-wavelength light during the night increased alertness even though long-wavelength light did not suppress melatonin levels [12,16].

The present results revealed that morning exposure to both short- and long-wavelength lights affected one measure of alertness. The fact that both, short- and long-wavelength light had the same effects on reducing alpha power suggests the possibility that mechanisms different than or in addition to those that are mostly sensitive to short-wavelength light (e.g. melatonin suppression) might be involved in mediating light-induced alertness observed in the present study. Moreover, since the short- and long-wavelength lights in this study were equated in terms of photopic illuminance, the cone photoreceptors (L- and M-cones) are probably participating in the alerting effects of light observed in this study.

Contrary to previous studies reporting that EEG activity in the theta frequency range was decreased [5,6,21], light did not significantly change power in the theta frequency range in the present study. The discrepancy between the current findings and these previous studies might be due to the degree of homeostatic sleep pressure that subjects were experiencing during the light exposure; in the previous studies, subjects were sleep deprived prior to or during the light exposure, whereas in the present experiment subjects have just had a night of sleep prior to coming to the laboratory. It has been previously suggested that power in the theta range increases when clear signs of drowsiness are exhibited in sleep-deprived subjects [17]. While subjects felt sleepier over the course of the experiment, as shown by the increase in theta power, light in the present study was only effective at reducing alpha power. These results suggest light may not be as effective at affecting a low homeostatic sleep drive, which is more likely to occur when subjects are not sleep deprived.

## Conclusions

The present study examined the effects of narrowband, colored light on human alertness in the early morning, when melatonin levels would be declining. We measured EEG activity during prolonged exposures to short- and long-wavelength light, and during darkness. EEG power in the alpha frequency range was significantly reduced during exposure to the short- and long-wavelength light, relative to the dark condition, after approximately 30 minutes. Since both short- and long-wavelength light affected EEG activity, these results suggest that the alerting effects of light in the early morning hours may be mediated by mechanisms independent of acute melatonin suppression, as has previously be demonstrated at night and during the afternoon. Further studies to understand better the pathways through which light can increase alertness should be investigated.

## Competing interests

The authors declare that they have no competing interests.

## Authors’ contributions

YO participated in the conception and the design of the experiment, data collection, data analyses and interpretation, and manuscript writing. MSR and MGF participated in the conception and the design of the experiment, data interpretation, and manuscript writing. All authors read and approved the final manuscript.
